# Are ChatGPT and large language models “the answer” to bringing us closer to systematic review automation?

**DOI:** 10.1186/s13643-023-02243-z

**Published:** 2023-04-29

**Authors:** Riaz Qureshi, Daniel Shaughnessy, Kayden A. R. Gill, Karen A. Robinson, Tianjing Li, Eitan Agai

**Affiliations:** 1 University of Colorado Anschutz Medical Campus, Aurora, CO USA; 2 PICO Portal, New York, NY USA; 3 University of Pittsburgh, Pittsburgh, PA USA; 4 Johns Hopkins University, Baltimore, MD USA

**Keywords:** Artificial intelligence, Large language models, Systematic review, Methodology

## Abstract

In this commentary, we discuss ChatGPT and our perspectives on its utility to systematic reviews (SRs) through the appropriateness and applicability of its responses to SR related prompts. The advancement of artificial intelligence (AI)-assisted technologies leave many wondering about the current capabilities, limitations, and opportunities for integration AI into scientific endeavors. Large language models (LLM)—such as ChatGPT, designed by OpenAI—have recently gained widespread attention with their ability to respond to various prompts in a natural-sounding way. Systematic reviews (SRs) utilize secondary data and often require many months and substantial financial resources to complete, making them attractive grounds for developing AI-assistive technologies. On February 6, 2023, PICO Portal developers hosted a webinar to explore ChatGPT’s responses to tasks related to SR methodology. Our experience from exploring the responses of ChatGPT suggest that while ChatGPT and LLMs show some promise for aiding in SR-related tasks, the technology is in its infancy and needs much development for such applications. Furthermore, we advise that great caution should be taken by non-content experts in using these tools due to much of the output appearing, at a high level, to be valid, while much is erroneous and in need of active vetting.

**Supplementary Information:**

The online version contains supplementary material available at 10.1186/s13643-023-02243-z.

In this commentary, we discuss ChatGPT and our perspectives on its utility to systematic reviews through the appropriateness and applicability of its responses to systematic review tasks and prompts. ChatGPT is a large language model (LLM) and artificial intelligence (AI) system designed by OpenAI (https://openai.com/blog/chatgpt/) to interact with people in a natural and conversational way [1]. Standard machine-learning (ML) algorithms are trained to provide responses or to make classifications or predictions given a specific input using large sets of data that have already been, or are actively, categorized by users [2, 3]. Likewise, LLMs are trained to predict language and writing based on large datasets of written language, thereby learning contextual clues and what might be expected or predicted following a set of words (i.e., a prompt) [3–5]. For example, OpenAI’s GPT-3.5 was trained on approximately 570 GB of text and is the original basis for ChatGPT [1, 6]. Depending on the prompt, LLMs can produce many different types of outputs. This has produced an explosion in the use of LLMs and ChatGPT, as well as creating controversy surrounding their applications [6].

Conducting a systematic review is a complex and arduous process that takes a great deal of expertise and time. It is not uncommon for reviews to take over 12 months to complete and require upwards of $100,000 in effort when considering the time spent on searching (by information specialists), screening, data extraction, analysis, interpretation, and writing by methodologists and content experts [7–9]. There are areas where ML and AI have already been introduced in the systematic review process with great success [10–13]. Given the recent attention around LLMs, like ChatGPT, and the resource burden of conducting a systematic review, we wanted to explore and critique the responses of ChatGPT to systematic review tasks. On February 6, 2023, developers of PICO Portal—an AI-assisted systematic review platform [10]—hosted a webinar to demonstrate a variety of tasks and elicit feedback on the ChatGPT output.

We “tested” ChatGPT by asking it to complete systematic-review tasks with a focus on tasks relevant to interpretation of language and not test whether ChatGPT could perform a task that is more data-specific, such as data extraction [14]. Other biomedical uses for LLMs have been suggested, including for data and text mining, particularly of clinical records, and aiding in medical education and clinical decision making [15, 16]. Our intent was to see whether this kind of language model could be used by someone who may wish to plan a systematic review, further develop a review question, or get help in drafting the search or analysis methods. A detailed description of our experience and a link to the webinar recording can be found in the SUPPLEMENT.

We found that ChatGPT could complete some systematic review tasks well, while others had clear room for improvement:

- In formulating a structured review question, creating eligibility criteria, and screening titles for relevance, ChatGPT’s output suggested the interpretation and contextualization of the prompt was appropriate. We felt the proposed criteria and selected articles could serve as a starting point for refinement depending on the complexity of the question.
- Having a ChatGPT generated PubMed search strategy, or an initial version, would be helpful to those who may not have access to an informationist in their resources. However, the proposed search strategy was unusable with multiple issues, including fabricating controlled vocabulary, that would not be apparent without expertise in search construction.
- ChatGPT is able to produce code in various programming languages and was able to create an outline of code for conducting a meta-analysis in Python and R. However, as with the search strategy, there were coding errors that required troubleshooting from a user with methodologic expertise.
- Synthesis and summary of multiple studies is a challenge but ultimately the most essential product of a systematic review. The time required to pick relevant information and create a summary is substantial, but there is potential for tools like ChatGPT to help begin these processes for reviewers. We found promise in the ability of the system to identify and summarize relevant information from a set of three abstracts. However, there were errors that suggest the technology is not yet ready for such a task.

In its current form, ChatGPT presents as an “uncanny valley” in research and information sciences: from a distance, the output mimics and passes as authentic; however, on closer inspection, it becomes apparent that it is not expertly formed material based on a depth of understanding of the systematic review process. A particularly strong limitation of the system is the lack of referencing appropriate and verifiable sources when asked for factual information. When we asked for references, we could not verify what it presented to us. This is a common occurrence as LLMs are designed to build a response using predictions and not by looking through literature to find real sources [17]. Indeed, when asked where it finds information and to search bibliographic databases, ChatGPT responds only that it cannot conduct any real literature retrieval.

With the model’s current capabilities, we anticipate that anyone attempting to use ChatGPT for providing verifiable and content/context-specific research will find that the recipient must have expertise in the subject matter. Unfortunately, this pre-requisite defeats the purpose of having an “intelligent” automation help with the tasks. It should be noted that other LLMs are being developed and entering the public domain, so ChatGPT may not perfectly reflect all LLMs. On March 15, 2023, GPT-4.0 was released for testing among OpenAI’s paid subscribers [18]. This new model purports to be more powerful than GPT-3.5 and better at recognizing and producing language and contextual cues in writing [18]. It should be noted that some minor testing of GPT-4.0 with similar questions showed a mild improvement in the summarization of three abstracts, but no additional improvements in systematic review task completion as far as we could discern. Additionally, there may be other systematic review tasks and use cases that we have not conceived and may elicit a more trustworthy and usable response from ChatGPT or other such systems. Furthermore, for better or worse, since generating a response in an LLM is not deterministic, the response will not be identical each time the same question is asked. We expect that there will be further advancements in the capabilities of these systems.

We know that the broader scientific community has concerns with the use of LLMs in research, and from our experience, we believe the systematic review community shares the same sentiments. Those in attendance during our demonstration posted many comments about ChatGPT and its application in education and research, primarily echoing concerns with the use of the technology and a large number of questions about its capabilities, limitations, internal processes, and output. Comments reflecting the uncertainty and hesitancy to use LLMs were also common, alongside the risks with non-expert use, as it was apparent that there was a requirement for content expertise in the various tasks. Many attendees posted links to resources and other tools to help perform the systematic review tasks we explored. There were also some comments on potential applications and areas for developing LLMs in the field of evidence syntheses and general positive and negative reactions from people about the utility of AI systems and LLMs in science. It is clear that discussion of the potential applications, challenges, and risks with integrating these technologies into the systematic review process need to happen and should take place in large, public forums. One group that is working towards addressing some of the questions and methodological issues such integration brings is the International Collaboration for the Automation of Systematic Reviews (ICASR) [19].

Despite the challenges with its use in systematic review tasks, ChatGPT was able to contextualize our questions and formulate responses that fit what we requested, which is encouraging for future development. In particular, we believe more attention should be given to accurately creating search strategies, as these utilize logic and rules (e.g., Boolean operators) and structured language in a way that should be more easily trainable than conversational language. Likewise, strengthening the ability of LLMs to take sections of text and identify relevant information for summary purposes could provide starting points for researchers or high-level summaries of results when time is limited (e.g., in a pandemic with hundreds of articles published daily that could contain relevant information to inform guidelines). Additionally, as the text writing and editing capabilities improve, the potential utility increases for these systems in polishing drafts of systematic reviews for authors who need help revising their writing [20].

In conclusion, we believe ChatGPT and other LLMs hold promise in being integrated into systematic reviews, but they are not yet able to be used with confidence in any way. We encourage others to attempt similar exploration and testing to understand the current limitations and capacity of ChatGPT and LLMs in the context of evidence synthesis.

## Supplementary Information

**Additional file 1: SUPPLEMENT.** Detailed description of our experience and a link to the webinar recording.

## Data Availability

Data sharing is not applicable to this article as no datasets were generated or analyzed during the current study.
