# Supplementary material for: Are ChatGPT and large language models “the answer” to bringing us closer to systematic review automation?
Source: Syst Rev. 2023 Apr 29;12:72. doi: 10.1186/s13643-023-02243-z (PMC10148473; doi:10.1186/s13643-023-02243-z)
Supplement: Supplementary file 1 — Additional file 1: SUPPLEMENT. Detailed description of our experience and a link to the webinar recording. [file 13643_2023_2243_MOESM1_ESM.docx]

**ChatGPT and Systematic Reviews – SUPPLEMENT**

Given the recent attention around large language models (LLMs) like ChatGPT and the resource burden of a conventional systematic review, we wanted to assess the responses of ChatGPT to systematic review tasks. On February 6^th^, 2023, developers of PICO Portal – an AI-assisted systematic review platform – hosted a webinar to demonstrate these tasks and elicit feedback on the ChatGPT output. This topic is of high interest and relevance to the community as 861 participants registered from 54 countries and 479 institutions, with 412 attending the webinar. This supplement summarizes ChatGPT’s responses to our prompts and discusses their applicability, correctness, and usefulness to reviewers according to our thoughts and those in attendance. A recording can be found at the following link:

[picoportal.org/2023/02/06/recording-chatgpt](http://picoportal.org/2023/02/06/recording-chatgpt/).

**HOW DOES ChatGPT RESPOND TO PROMPTS FOR SYSTEMATIC REVIEW METHODS?**

Systematic review requires deep understanding of language and context as an important part of identifying and synthesizing relevant evidence for a user. As LLMs are built to recognize contextual cues in large volumes of text, our focus was on review tasks that an LLM might be able to achieve; for example, creating a structured review question based on an intervention, condition, and outcome of interest, or identifying which titles might be relevant given a specific question. We focused on tasks relevant to the interpretation of language and did not test whether ChatGPT could perform a task that is more data-specific, such as data extraction.^1^ Other biomedical uses for LLMs have been suggested, including data and text mining, particularly of clinical records, and aiding in medical education and clinical decision making.^2,3^ Our intent was to see whether this kind of language model could be used by someone who may wish to plan a systematic review or further develop a review question.

To this end, we asked ChatGPT the questions in **Box 1** related to systematic review methodology; some are more task-based, while some are more philosophical and general review methodology focused.

**[INSERT BOX 1]**

**Formulating a systematic review question**

In Questions 1 & 2, regarding the development of a question and related eligibility criteria, the output from ChatGPT suggested an appropriate interpretation of the task and the desired structure for a typical systematic review. The question output followed the PICO framework, providing a reasonable first draft of a question **(Figure 1a)**. In creating eligibility criteria, ChatGPT provided criteria for the PICO elements while also including study design, language, publication date, study participants, and dose **(Figure 1b)**. While the criteria for participants and dose could respectively be combined with those for the Population and Intervention, and restrictions on language and publication date may be inappropriate in some contexts, the proposed criteria could serve as a starting point for further development depending on the complexity of the question.

**[INSERT FIGURE 1a/1b]**

**Writing a systematic review search strategy**

When tasked with developing a search strategy for PubMed, ChatGPT’s output matched the structural components of a search; however, inspection revealed major limitations. The ChatGPT developed search string was:

*(("Chocolate"[MeSH Terms] OR "Chocolate"[All Fields]) OR ("Flavonones"[MeSH Terms] OR "Flavonones"[All Fields]) OR ("Cocoa"[MeSH Terms] OR "Cocoa"[All Fields])) AND ((("Hypertension"[MeSH Terms] OR "Hypertension"[All Fields]) OR ("High Blood Pressure"[MeSH Terms] OR "High Blood Pressure"[All Fields])) OR ("Arterial Hypertension"[MeSH Terms] OR "Arterial Hypertension"[All Fields]) OR ("Essential Hypertension"[MeSH Terms] OR "Essential Hypertension"[All Fields])) AND ("Clinical Trial"[Publication Type] OR "Randomized Controlled Trial"[Publication Type]).*

At its face, the search appears to utilize required aspects that one would expect in a systematic review search. Both controlled vocabulary terms (i.e., MeSH and Publication Type terms) and keywords are used and combined with the Boolean operator “OR”, and there are multiple concepts (Intervention, Population, Study type) that are joined with “AND”. However, upon inspection by an information specialist or someone experienced in systematic reviews, there are many problems that would make it unusable. Among the issues:

1. ChatGPT fabricated pseudo-MeSH Terms that resemble genuine ones that either return no results (e.g., “Cocoa” is not a MeSH term, the correct term is “Cacao”), or redirect to existing MeSH terms creating unnecessary duplication (e.g., “High blood pressure” is not itself a MeSH term and is a synonym or entry term for the real MeSH: “Hypertension”)
2. ChatGPT created other MeSH terms that do not have a potential match (e.g., no term exists for “Arterial hypertension” or “Essential hypertension”)
3. The study type concept has applied a filter for clinical trials but it is not validated and will limit the returned records, excluding many potentially eligible trials that are not tagged with those two Publication Types.

To the untrained eye, this search appears to meet the requirements of a systematic review; however, running it in PubMed does not retrieve any results. If it did, the sensitivity and specificity of the search would be unacceptable for a systematic review. This particular task has also been tested by others and was found to have similar problems.^4^

**Screening titles for eligibility**

There are existing models used in systematic review software that can help identify potentially relevant study records based on how screeners make eligibility decisions. However, we were curious about the potential for an LLM to recognize relevant study records based on a single review question without any question-specific training from screeners. We fed ChatGPT 27 titles of trial registrations from clinicaltrials.gov and asked for the relevant titles given our same question of interest in previous examples. The system identified 9 titles (9/27, 33%) as relevant, eight of which (89%) we agreed would have met the inclusion criteria for the systematic review. The system did not include articles that were clearly unrelated, but it missed two articles (2/9, 22%) that could potentially be related, likely because these articles did not have the exact language for Population, Intervention, and Outcomes specified in our question. Considering this result within the context of not having undergone eligibility training, the model's performance was promising. While not accurate enough to replace trained methodologists, there is a potential utility for this kind of language model in performing an initial “screening” to see whether the drafted search captures relevant records and which might be of greatest interest.

**Setting up code and data for analyses**

When a meta-analysis is appropriate in a systematic review, reviewers typically use programs such as RevMan, R, SAS, or Stata. While R and Python are open access, they have learning curves and preparing the data for analysis requires specific knowledge of the program. We asked ChatGPT to write code for two different systems for conducting a meta-analysis: Python and R (**Figure 2a and 2b**). The R code output from ChatGPT sets up an example dataset and then makes use of the existing codes for meta-analyses. ChatGPT did make a mistake, however, by failing to include a necessary line of code, thereby preventing the overall code from running. Regardless of whether the code runs without troubleshooting, time and expertise are required to identify potential errors and adjust the code. The generated Python script was of interest as ChatGPT created the code for the meta-analysis and a forest plot to present the estimates. While the axes on the plot are reversed from how meta-analyses are typically presented, and the summary estimate and confidence interval are plotted in an unconventional way, the graph is interpretable (**Figure 2a**). The challenge, however, is that the system wrote its own code for the actual statistical combination, and without statistical expertise, it would be impossible to judge if the formulas and combinations are valid.

**[INSERT FIGURE 2a/2b]**

While the writing of functioning code is impressive, given the many assumptions that need to be checked for a meta-analysis, this is an area that should be left to someone with methodological expertise on the systematic review team.

**Informing and guiding review methods**

ChatGPT was also asked to answer methodology questions that might arise while conducting a systematic review. Specifically, (i) whether a meta-analysis should be done given clinical and methodological heterogeneity between studies and (ii) whether summaries of evidence, like those using the GRADE framework, should include specific details from a synthesis, such as point estimates. ChatGPT provided acceptable answers for these questions. It is tempting to suggest that LLMs could be useful to people who may not know how to find answers to such questions as a substitute for searching Google or literature databases for relevant methods papers. However, ChatGPT is known to make up responses to fulfill a prompt based on its predictive models, and a non-expert may not know whether a response is correct.^5–7^

**Summarizing multiple studies**

Lastly, the most important and arguably challenging task of systematic review is the actual synthesis of multiple studies. True synthesis requires an understanding of each study’s results and context to qualitatively summarize the findings in a cohesive and valid way. The first step to synthesis is to accurately summarize each study. To test whether ChatGPT could synthesize results from multiple studies, we asked for a summary of the findings from three abstracts and a recommendation. While recommendations for practice are typically an aspect of guidelines, as opposed to systematic reviews, many systematic reviews do include an interpretation of the results based on their findings. The response is shown in **Figure 3** and shows the model’s limitations, although there is promise with future development.

**[INSERT FIGURE 3]**

The interpretation of the findings and clinical implication was acceptable given that no strong recommendation should be made due to the heterogeneity of the evidence across the studies. The implication and call for more research was also acceptable and a common conclusion in systematic reviews. Regarding the summary, ChatGPT accurately identified relevant results for the first two abstracts and presented them as summary points. However, the model incorrectly pulled a “result” from the third abstract’s background. The inherent limitation of these types of language models, as noted in media stories about ChatGPT and as acknowledged on OpenAI’s website, is that the models will sometimes make up data or responses to prompts that sound good and fit the question/prompt, but are not actually real or correct.^7^ The model does not tell the user when it does this and presents no form of confidence around its responses when it does make an inference and presents something as “a fact”. This limitation is a major hurdle to be overcome in expanding the utility of these models for research. Once again, a challenge to using this new technology is that content expertise is required to interpret the response and know whether it is appropriate. Someone naive to the limitations of ChatGPT, systematic review methods, or the specific topic of the review question, could be misled into believing the output is correct when it may not be the case.

**WHAT DO PEOPLE THINK OF ChatGPT FOR CONDUCTING SYSTEMATIC REVIEWS?**

The webinar was attended by over 400 people from around the world. The occupations and experiences of the attendees are unknown, however, over 400 institutions were represented in the 800 registrations, suggesting that attendees were mostly affiliated with academic institutions and likely had some familiarity with the concept of evidence synthesis and systematic reviewing.

During the webinar, the chat received over 150 comments, not including general agreements or reactions to comments made by other attendees. We read the comments and categorized them broadly based on the content (**Box 2**). There were many comments about ChatGPT and its application in education and research, primarily echoing concerns with the use of the technology and a large number of questions about its capabilities, limitations, internal processes, and output. Comments reflecting the uncertainty and hesitancy to use LLMs were also common, alongside the risks with non-expert use, as it was apparent that there was a requirement for content expertise in the various tasks. Many attendees posted links to resources and other tools to help perform the systematic review tasks we explored. There were also some comments on potential applications and areas for the development of LLMs in the field of evidence syntheses and general positive and negative reactions from people about the utility of AI systems and LLMs in science.

**REFERENCES**

1. RobotReviewer. RobotReviewer - Automating evidence synthesis. RobotReviewer. Published 2023. www.robotreviewer.net

2. Kung TH, Cheatham M, Medenilla A, Sillos C, De Leon L, Elepaño C, Madriaga M, Aggabao R, Diaz-Candido G, Maningo J, Tseng V. Performance of ChatGPT on USMLE: Potential for AI-assisted medical education using large language models. *PLoS Digit Heal*. 2023;2(2):e0000198. doi:10.1371/journal.pdig.0000198

3. Lewis P, Ott M, Du J, Stoyanov V. Pretrained language models for biomedical and clinical tasks: Understanding and extending the state-of-the-art. In: *Proceedings Ofthe 3rd Clinical Natural Language Processing Workshop*. ; 2020:146-157. doi:10.18653/v1/2020.clinicalnlp-1.17

4. SRLibrarianProblems. @SRLibrarianProblems. Twitter. Published 2023. Accessed February 10, 2023. twitter.com/SRLibProblems

5. Shen Y, Heacock L, Elias J, Hentel K, Reig B, Shih G, Moy L. ChatGPT and other large language models are double-edged swords. *Radiology*. 2023;1. doi:10.1148/radiol.230163

6. Tamkin A, Ganguli D. How large language models will transform science, society, and AI. *Stanford Univ Human-Centered Artif Intell*. Published online 2021. https://hai.stanford.edu/news/how-large-language-models-will-transform-science-society-and-ai

7. OpenAI. ChatGPT: Optimizing language models for dialogue. OpenAI. Published 2023. Accessed February 6, 2023. https://openai.com/blog/chatgpt/

**BOXES, TABLES, AND FIGURES**

**Box 1.** Set of questions asked of ChatGPT

| **Question** |
| --- |
| 1. Please structure a systematic review question using the PICO framework to explore the effects of dark chocolate on blood pressure among people with hypertension. |
| 1. Write eligibility criteria for the following systematic review question: What is the effect of chocolate on blood pressure among people with hypertension? |
| 1. Create a PubMed query for a systematic review using keywords and MeSH terms to find relevant clinical trials. The intervention concept should be related to “chocolate”, “flavonones”, and relevant synonyms. The population should be related to “hypertension”, “high blood pressure”, and include other synonyms. |
| 1. From the following 27 titles, tell me which ones are relevant if I'm interested in the effectiveness of chocolate on reducing blood pressure:   *"Effects of Cocoa on Ambulatory Blood Pressure and Vascular Function in Patients With Stage I Hypertension"*  *"Acute Hemodynamic Effects of Cocoa Polyphenols in Subjects With Hypertension and Optimal Blood Pressure"*  *"Effects of Cocoa Intake on Blood Pressure in Elderly Individuals With Mild Hypertension"*  *"Effects of Dark Chocolate on Insulin Sensitivity in People With High Blood Pressure"*  *"Effect of Cocoa Consumption in Cardiovascular and Immune Parameters"*  *"Effects of Acute Ingestion of Dark Chocolate on Endothelial Function in Hypertensive Patients"*  *"Effect of a Polyphenol Rich Diet on Vascular and Platelet Function"*  *"Controlled Clinical Trial to Determine the Effective Dose of Cocoa in Lowering Blood Pressure"*  *"CoCo: Colour Coded Blood Pressure Control"*  *"Effect of Different Dosages of Dark Chocolate on Arterial Blood Pressure in Cardiovascular High-risk Patients"*  *"Chocolate Consumption in Healthy Pregnant Women Trial"*  *"The Effect of Cocoa Flavonoids on Blood Pressure"*  *"Cocoa, Polyphenols, and the Kidney in Healthy Subjects and in Subjects With Hypertension and Diabetes Mellitus"*  *"Flavanol-Rich Cocoa and Cerebral Blood Flow"*  *"The Effect of Green Tea or Cocoa Extracts in Endothelial Dysfunction in Patients With Diabetes Mellitus"*  *"Effects of Dark vs. White Chocolate on the Postprandial Increase in Portal Pressure in Cirrhosis"*  *"(-)-Epicatechin and Pulmonary Arterial Hypertension"*  *"Effects of Flavanoids in Essential Hypertension, Type 2 Diabetes and Healthy Persons"*  *"Study on the Effects of Epicatechin and Quercetin Supplementation on Vascular Function and Blood Pressure (FLAVO)"*  *"Effects of XoÃ§ai Antioxidant Supplements on Atherosclerosis Risk Factors"*  *"The Effects of Dark Chocolate on Blood Pressure in Individuals With Mildly Elevated Blood Pressure"*  *"Effect of an Apple Polyphenol Extract on Brachial Artery Flow-mediated Vasodilatory Function"*  *"Consumption of Chocolate in Pregnant Women."*  *"Irbesartan/Hydrochlorothiazide (HCTZ) Combination Therapy as First Line Treatment for Severe Hypertension"*  *"A Study of the Effectiveness and Safety of Ramipril in the Treatment of Hypertension in Children and Adolescents"*  *"Grain Exercise Trial - Whole Grain Versus no Grain With or Without Physiotherapy-promoted Exercise for Waist Loss"*  *"Physiological Effects of Grape Seed Extract in Diastolic Heart Failure"* |
| 1. Write a Python program that uses meta-analysis to find the average effect (relative risk of an outcome) for an intervention versus a comparator from multiple studies and plot the individual and overall estimates and their 95% Confidence Intervals in a forest plot.   Write an "R" program with sample data that uses meta-analysis to find the average effect (relative risk of an outcome) for an intervention versus a comparator from multiple studies and plot the individual and overall estimates and their 95% Confidence Intervals in a forest plot. |
| 1. I am conducting a systematic review. If I have high heterogeneity across my included studies, should I perform a meta-analysis? |
| 1. Following GRADE for systematic reviews, should summary estimates of effects be included with recommendations? |
| 1. Using the following abstracts from three different studies, create a summary of the findings and a recommendation for practice:   *“Context: Regular intake of cocoa-containing foods is linked to lower cardiovascular mortality in observational studies . Short-term interventions of at most 2 weeks indicate that high doses of cocoa can improve endothelial function and reduce blood pressure (BP) due to the action of the cocoa polyphenols, but the clinical effect of low habitual cocoa intake on BP and the underlying BP-lowering mechanisms are unclear. Objective: To determine effects of low doses of polyphenol-rich dark chocolate on BP. Design, Setting, and Participants: Randomized, controlled, investigator-blinded, parallel-group trial involving 44 adults aged 56 through 73 years (24 women, 20 men) with untreated upper-range prehypertension or stage 1 hypertension without concomitant risk factors. The trial was conducted at a primary care clinic in Germany between January 2005 and December 2006. Intervention: Participants were randomly assigned to receive for 18 weeks either 6.3 g (30 kcal) per day of dark chocolate containing 30 mg of polyphenols or matching polyphenol-free white chocolate. Main Outcome Measures: Primary outcome measure was the change in BP after 18 weeks . Secondary outcome measures were changes in plasma markers of vasodilative nitric oxide (S-nitrosoglutathione) and oxidative stress (8-isoprostane), and bioavailability of cocoa polyphenols. Results: From baseline to 18 weeks, dark chocolate intake reduced mean (SD) systolic BP by -2.9 (1.6) mmHg (P <.001) and diastolic BP by -1.9 (1.0) mmHg (P <.001) without changes in body weight, plasma levels of lipids, glucose, and 8-isoprostane . Hypertension prevalence declined from 86% to 68% . The BP decrease was accompanied by a sustained increase of S-nitrosoglutathione by 0.23 (0.12) nmol/L (P <.001), and a dark chocolate dose resulted in the appearance of cocoa phenols in plasma . White chocolate intake caused no changes in BP or plasma biomarkers. Conclusions: Data in this relatively small sample of otherwise healthy individuals with above-optimal BP indicate that inclusion of small amounts of polyphenol-rich dark chocolate as part of a usual diet efficiently reduced BP and improved formation of vasodilative nitric oxide.”*  *“BACKGROUND: Several studies have shown that cocoa and cocoa-containing foods have the potential to lower blood pressure and improve endothelial function . Most of the studies reporting the beneficial effects of dark chocolate on blood pressure have been short (≤ 4 weeks). The aim of the present 8-wks (weeks) study was to assess the effects of regular consumption of dark chocolate during a reduced snack consumption intervention on blood pressure and other cardiovascular risk factors in mildly hypertensive individuals. DESIGN: This was a randomized, controlled, cross-over trial involving 22 adults (8 women, 14 men), aged 33-64 y, BMI 27.7 ± 3.7 kg/m (2) with mild hypertension. During the intervention period (8-wks) the participants reduced the intake of habitual snacks and replaced them with dark chocolate (49 g/day). In the control period, they only reduced the snacks without any added chocolate. Data (blood lipid profile, glucose, insulin, 24 h blood pressure) was collected in the beginning and end of both periods (intervention and control), and some variables also in the run-in and run-out periods (weight, body fat percentage, blood pressure, arterial stiffness index, diet and physical activity). RESULTS: Daily consumption of dark chocolate had no effects on 24 h blood pressure, resting blood pressure (mean ± SD, pre 142 ± 11.5/89 ± 8.4 mmHg vs. post 142 ± 14.2/88 ± 9.4 mmHg in systolic and diastolic blood pressure, respectively) or arterial stiffness (mean ± SD, pre 7.68 ± 0.88 vs. post 7.76 ± 0.89). Weight was reduced by 1.0 ± 2.2 kg during the control (reduced snack only) period, but was unchanged while eating chocolate (p <0.027 between the treatments). CONCLUSION: The data collected in this study indicates that inclusion of dark chocolate daily in the diet had no significant effects on blood pressure or other cardiovascular risk factors during a reduced snack period.”*  *“Regular cocoa consumption has been shown to improve blood pressure (BP), insulin sensitivity, and lipid levels in patients with type 2 diabetes (T2D), using up to 100 g of chocolate or 54 g of cocoa. These effects, attributed to cocoa flavanols, would be beneficial for patients with T2D if they could be achieved by a usual serving size of flavanol-rich cocoa. Forty-two hypertensive patients with T2D (stable pharmacological treatment, with good adjustment for glucose metabolism, lipids, and BP) ingested capsules with 2.5 g/day of a flavanol-rich cocoa or cocoa-free capsules for 12 weeks in a double-blinded, randomized, placebo-controlled study with parallel group design . Participants had to maintain diet, lifestyle, and medication . Before and after intervention, fasting blood samples were collected; BP and nutritional status were investigated . Cocoa treatment did not affect BP, nor glucose metabolism (glucose, HbA (1c), insulin, HOMA-IR) and lipids (triglycerides, total cholesterol, low-density lipoprotein cholesterol, high-density lipoprotein cholesterol). Body weight, fat mass, and nutrient supply remained unchanged . Changes in the placebo group did not occur . Regular intake of a usual serving size of flavanol-rich cocoa does not improve cardiometabolic parameters in stably treated patients with T2D and hypertension. As the medication modulates partly the same targets as cocoa flavanols, future studies should focus on the preventive effect of cocoa against diabetes and other cardiometabolic diseases in individuals with preexisting abnormalities that do not require any pharmacological treatment.”* |


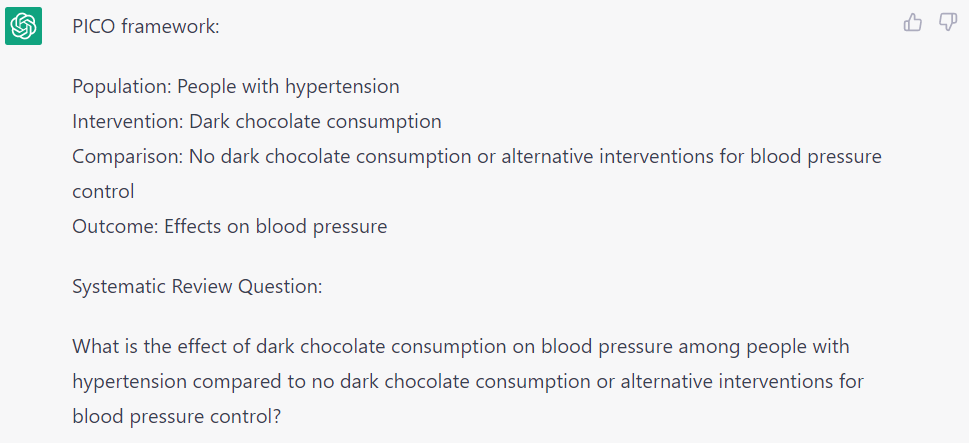


**Figure 1a**. ChatGPT response to Question 1.


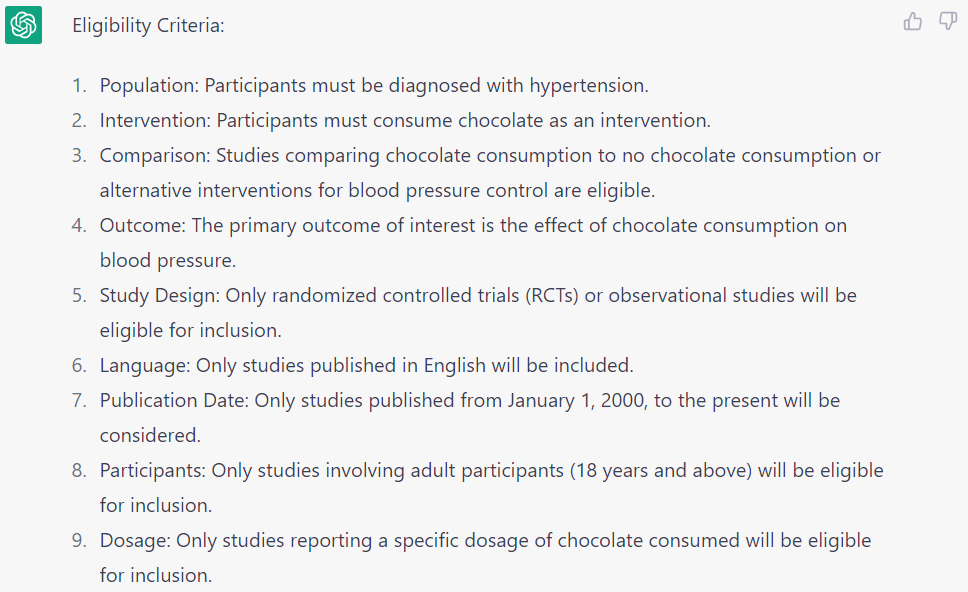


**Figure 1b.** ChatGPT response to Question 2.


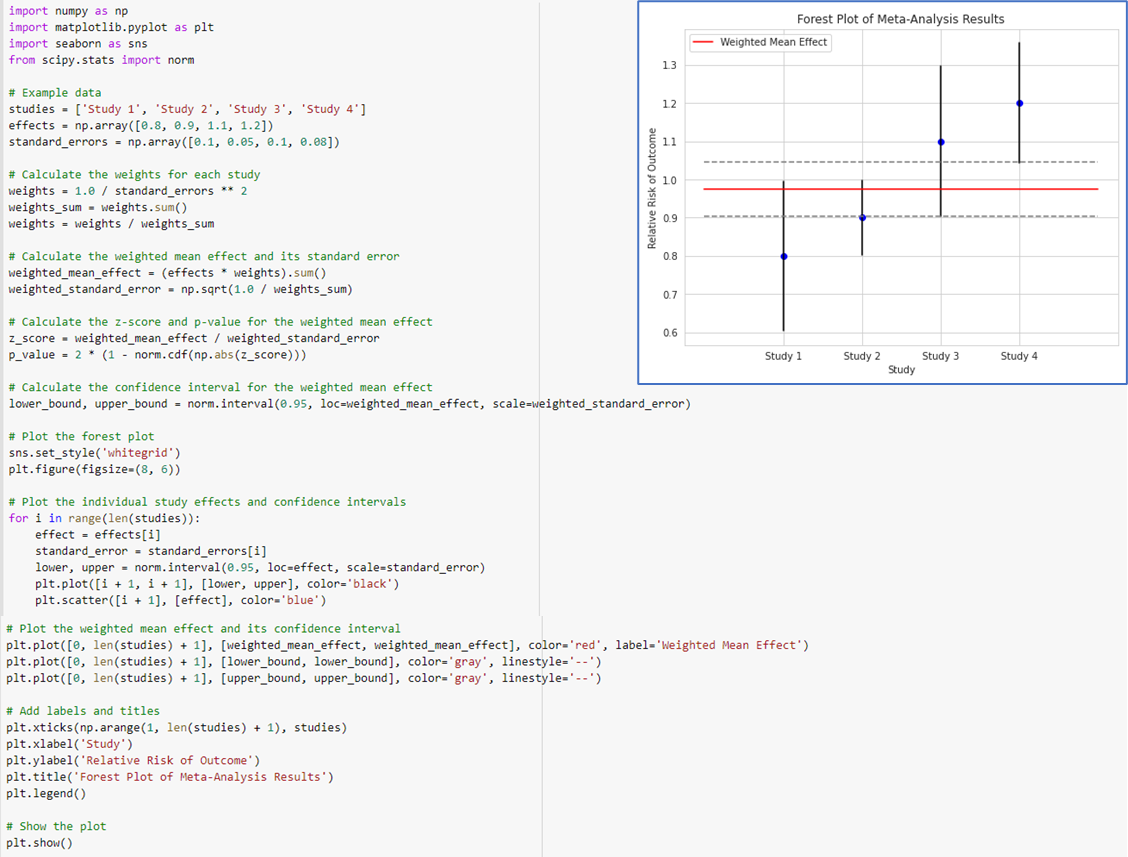


**Figure 2a.** Python code to perform meta-analysis


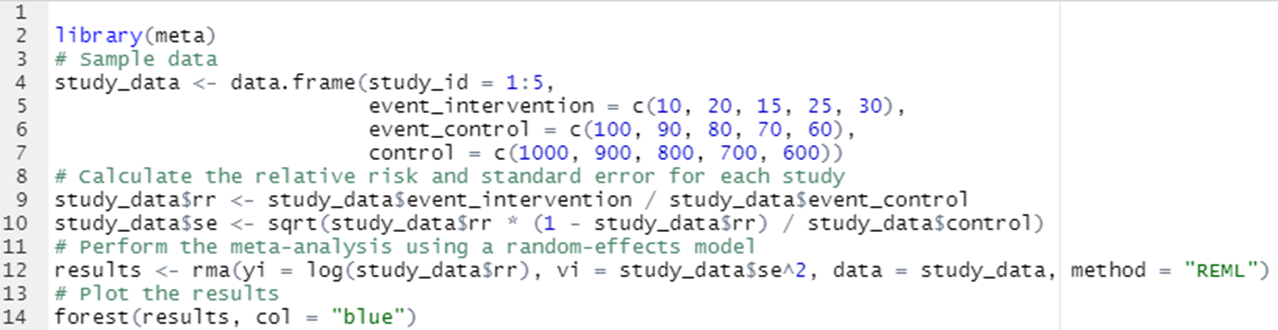


**Figure 2b.** R code to perform a meta-analysis


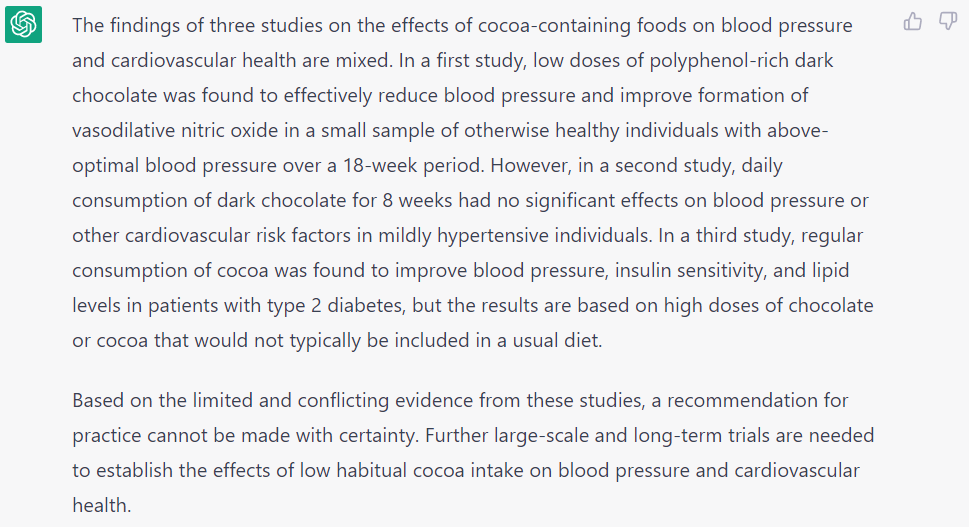


**Figure 3**. ChatGPT response to Question 8

**Box 2.** Themes in chat from attendees

| - Comments about the application in education and research; primarily concerns with students using and either not learning or learning incorrectly; also concerns with plagiarism and authorship attribution - Questions about the capabilities and limitations of ChatGPT in general (i.e., where is it pulling information; can it do X, Y, Z) - Concerns about ChatGPT processes and output (i.e., making up content to fulfill a prompt: references, MESH Terms, general content) - Comments on the risk of non-expert use (i.e., not recognizing problems with the ChatGPT search strategy) - Comments about uncertainty and hesitancy to use LLMs because of their lack of transparency and users’ lack of understanding of how the system is working - Links and resources to other tools/resources that do systematic review tasks - Potential applications and areas for development - General positive and negative reactions from people in the utility of AI systems and large language models |
| --- |
